# The effect of group size, age and handling frequency on inter-male aggression in CD 1 mice

**DOI:** 10.1038/s41598-020-59012-4

**Published:** 2020-02-10

**Authors:** Paulin Jirkof, Natalie Bratcher, Letty Medina, Donna Strasburg, Paige Ebert, Brianna N. Gaskill

**Affiliations:** 1 Division of Surgical Research, University Hospital Zurich, Zurich, Switzerland; 2 Office of Animal Welfare, AbbVie, North Chicago, USA; 3 Comparative Medicine, AbbVie, North Chicago, USA; 4 Department of Animal Science, Purdue University, West Lafayette, USA

**Keywords:** Social behaviour, Feeding behaviour, Animal behaviour

## Abstract

Aggression in male mice often leads to injury and death, making social housing difficult. We tested whether (1) small group size, (2) early age of allocation to a group decreases aggression and 3) manipulation increases aggression in male mice. A 14wk study was performed to assess the following conditions in male CD-1/ICR mice: group size (1, 2, or 3), age at grouping (5 or 7wks), and manipulation (daily scruffing or minimal weekly handling). Wounds, body weights, food consumption, nest scores, sucrose consumption, fecal corticosterone and blood for hematology were collected. At the end of the study, mice were euthanized and pelted to assess wounding with the pelt aggression lesion scale (PALS). No signs of acute or chronic stress were observed in any of the groups. Trio housed mice showed less bite wounds than pair housed mice. In general, mice in larger groups ate less but weighed more. Individually housed mice, however, had high nest scores, low body weights, and increased sucrose and food consumption. These results suggest that even when nesting material is provided, individual mice may be experiencing thermal stress. Based on this data, CD-1 mice can successfully be housed for up to 14wks and groups of 3 may be the best for reducing even minor levels of aggression (i.e. wounding).

## Introduction

The laboratory mouse, like its wild ancestor, the house mouse, is considered to be a social species. International legislation, such as the EU Directive 2010/63/EU and the *Guide for the Care and Use of Laboratory Animals*, stipulate or recommend social housing for mice whenever possible. Housing female mice in harmonious, same-sex, groups is simple and advantageous^1^. However, male mice often display inter-male aggression, which presents a welfare concern and often prevents or discourages social housing of male mice. This presents a significant challenge for those working with male mice and can introduce experimental variability when male mice are single housed and female mice are socially housed. The aggressive behavior seen in male mice can be explained by looking at the social structure of wild house mice, or mice housed under naturalistic or semi-natural conditions. Depending on the availability of resources, a single male mouse might defend a territory against other male mice, or share a territory with a few other male mice; these latter male groups are organized by a dominant-subordinate relationship. Several males might also live in isolation or in loose male groups in between defended territories^2–4^. Therefore, dominance behavior, aggression and fighting in males can be considered normal male mouse behavior. Unfortunately, the housing conditions and care procedures in modern laboratory husbandry may actually exacerbate aggression. Laboratory mice are housed in same sex groups in small enclosures, are often mixed at various ages with unfamiliar cage mates, undergo experimental procedures and face frequent disruption of odor cues due to cage cleaning procedures. All of these common procedures may be stressful and may bear the potential to lead to escalated fighting, severe injury, or even death. See^5–7^ for a comprehensive discussion.

The issue of aggression in groups of male mice typically leads to two management solutions: (1) male mice are socially housed until a certain degree of aggression occurs and are then separated and housed individually until the end of the study^8^ or (2) males are individually housed for the entire duration of a study^9^. The first approach has several detrimental consequences when fighting occurs, as animals might be severely injured or killed. This raises significant welfare concerns and leads to the dismissal of the affected animals from the study, which may increase total animal numbers and costs. At the very least, repeated, negative social interactions can be a profound source of stress for male mice^10^, again raising welfare concerns and possibly affecting variability in experimental results^7^. Therefore, males are often housed individually rather than in groups, especially in long-term studies, which may last for several months to years.

For obvious reasons, single housing of male mice has been topic of debate for several decades. Nevertheless, the findings from studies analyzing the actual effects of individual housing on male mouse well-being are ambiguous. Several studies using behavioral data, adrenal tyrosine hydroxylase activity or excretion of corticosterone metabolites, have found no support that group housing is superior to solitary housing. However, short increases of corticosterone metabolite levels have been observed immediately after separation^11–13^. Unfortunately, only one of these previous studies conducted in-depth behavioral observations along with physiological measures^12^. Therefore, it is unclear if singly housed mice were compared to harmonious or aggressive male groups. A fact that makes animal welfare assessment ambiguous since stress hormones do not indicate emotional valence. On the contrary, several studies show that individual housing may affect sympathetic neurotransmission^14^ as well as memory, emotionality, anxiety and activity in behavioral tests^15,16^ which may hint at a reduction in animal well-being. For example, Kalliokoski^17^ found that single housing of male mice led to more marked hypothermia after a selective serotonergic agonist challenge. However, even if parameters are unaffected in naïve individually housed males, at least one study found that solitary males might be more sensitive to stress, as seen in higher blood corticosterone levels compared to socially housed males^18^. Additionally, male mice benefit from the presence of same-sex cage mates during challenging health conditions^19,20^. Despite the controversy of the available studies, if male mice, irrespective of dominance status, are given a choice, they seem to prefer social contact with other males over being alone^6,21,22^.

Despite the complexity, the use of male mice in research is essential for ethical, economic, and scientific reasons^23^. While certain research questions directly target specific phenomena that occur in males only, the importance of using both sexes in animal experimentation has received significant attention and might have high relevance for the translation of experimental results to both male and female human patients.

To achieve harmonious social housing of male mice, several efforts have been made to improve male mouse housing routines. Male mice are often weaned into single-sex groups and are later relocated to smaller experimental groups. It has been discussed whether kinship and early age of grouping (i.e. before puberty) might reduce development of aggression between cage mates^6,24^. While anecdotal reports^9^ support these claims, available data appears to be ambiguous. Bartolomucci *et al*.^25^ report, for example, higher levels of aggression in groups of male mice when grouped before puberty compared to grouping after puberty. However, other studies show no difference in the preference of males for related or unrelated male cage mates^22^, and severe aggression between male siblings is well known^26^. Comparisons of the effects of different group sizes on male aggression yield more consistent data, with smaller group sizes (i.e. 3–5) resulting in more stable and less aggressive groups^6,27–31^. Nevertheless, at least one study^12^ reports no effect of group size on aggression in male mice. While environmental enrichment items might bear the risk of becoming a defended resource, which can increase aggression in male mice^32–34^, the use of nest building material, and especially the transfer of nesting material during cage change or the use of burrow-like, cage dividing structures has been shown to be effective in reducing aggression^28–30,35^. Additionally, it has been suggested that repeated manipulation, as typical in many research settings (e.g. restraint and injection or gavage), has the potential to increase aggression^24,36^. Reducing overall stress levels, where possible, might be beneficial for male cohesion^24^.

Despite anecdotal reports on the successful use of the combinations of factors named above in long-term toxicological research^9^, neither original data, nor details on the housing conditions that prove beneficial effects have been published. Therefore, the aim of this experiment was to test the effects of different housing conditions and husbandry procedures on aggressive behavior and well-being of male mice. We hypothesized that 1) smaller groups size decreases aggression, 2) earlier age of allocation to an experimental group/cage decreases aggression and 3) handling increases aggression in male mice. Male CD-1/ICR mice, a commonly used outbred mouse stock that has previously been described as aggressive^37^, were housed under different housing conditions (single, pair or trio housing; group allocation at 5 or 7 weeks of age; daily or weekly handling) for 14 weeks. Behavioural, clinical, endocrinological and hematological indicators were assessed during the course of the study to test our hypotheses.

## Results

### General clinical observations and necropsy

During daily observations and necropsy abnormalities, injuries or symptoms of illness were found in 15 of 120 (12.5%) animals. Twelve (10%) of these animals had preputial adenitis or preputial swelling of varying sizes or urinary scalding/irritation. This is relatively common in male mice and results from a bacterial infection^38,39^ but is not considered a serious illness. As the rate was slightly higher than expected^39^ we included “illness” as a blocking factor in our analyses in case it might explain data variability. This blocking factor was only found to be significant in the body weight analysis. In addition to the adenitis, one mouse had a malformation of the sternum, one a severe malformation of the hind limb nails and one animal developed a mass and showed signs of dehydration within a few days of arrival. Only 2 cages (an individually housed, 5 week old, scruffed mouse and a trio cage of 5 week old, minimally handled mice) were euthanized prematurely due the development of these symptoms.

### In-cage social interaction

#### Daily observations (occurrence of fighting)

During the first half of the experiment (week 0–6), only two wounded animals were observed: One of those animals was assigned to pair housing, allocated at 5 weeks of age, with scruffing, while the other mouse was assigned to trio housing, combined 7 weeks of age, with minimal handling. Both were identified as having minor wounds in week 3 of the study. In the second half of the study (week 7–13), the number of observed wounds increased in few groups. Repeatedly, one cage consisting of pair-housed males (5 weeks, minimal handling) showed wounds around week 12 and one pair of the same experimental group at week 13. One animal in a cage consisting of a pair (7 weeks, minimal handling) was found wounded in week 13 and finally one animal in a trio (5 weeks, minimal handling) was recorded as wounded repeatedly in week 13. In general however, no severe wounding was found and no animals had to be separated or euthanized because of escalated fighting.

Animals were rarely found out of the nest (approximately 2.2% of observations), in which case data was not analyzed.

#### PALS

**Figure 1:**
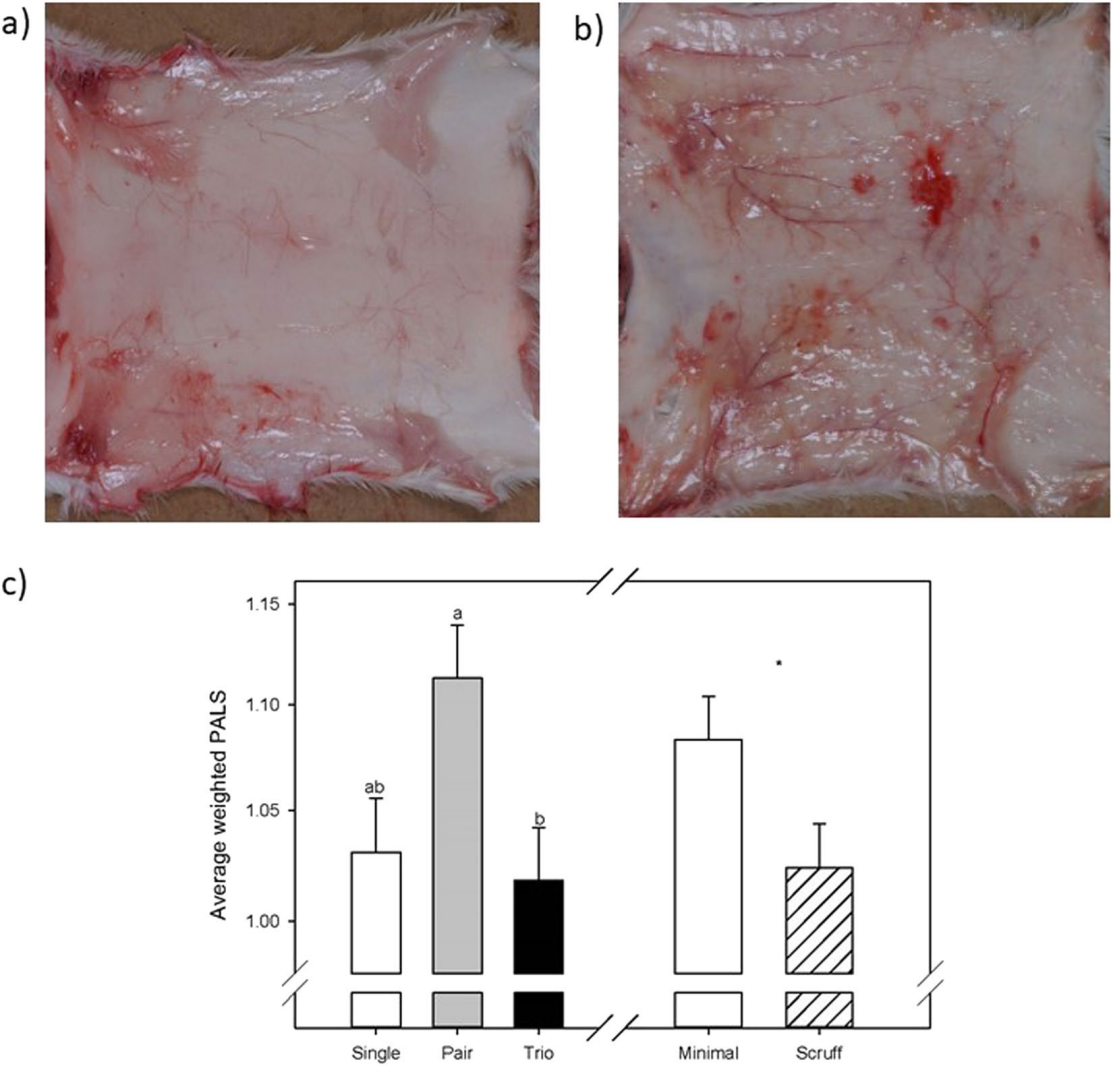
(**a**) False positive PALS (Photo ID 4885, animal ID 102, single, 7 weeks, minimal handling). Spots on the inside right flank, some small spots on the back and neck area. (**b**) True positive PALS (Photo ID 4766, animal ID 7, pair, 5 weeks, minimal handling). Spots everywhere but not the tail area, vessels, no pigmentation like seen in B6. (**c**) Average weighted PALS LSM ± SE are plotted. Data on the y-axis is represented as a log_10_ transformed scale but values have been backtransformed to be meaningful in the real world. Different letters above bars indicate significant differences by post-hoc Tukey tests (P < 0.05).

Group size affected the cage average PALS significantly (F_2_,_47_ = 4.3; P = 0.019; Fig. 1c). Pair housed mice had higher PALS (i.e. overall wounding) than trios (P < 0.05). The PALS of individually housed mice were not significantly different from pair or trio housed mice (P > 0.05). Minimally handled mice also had significantly higher PALS than mice scruffed daily (F_1_,_47_ = 4.3; P = 0.042; Fig. 1c). Of the 25 mice with a positive PALS, only 4 were identified by daily cage side observation as wounded.

We observed some false positive PALS in individually housed animals which were likely due to the hemorrhage in the neck area of many animals from the secondary method of euthanasia (Fig. 1a).

### Mental well-being

#### Sucrose consumption

**Figure 2:**
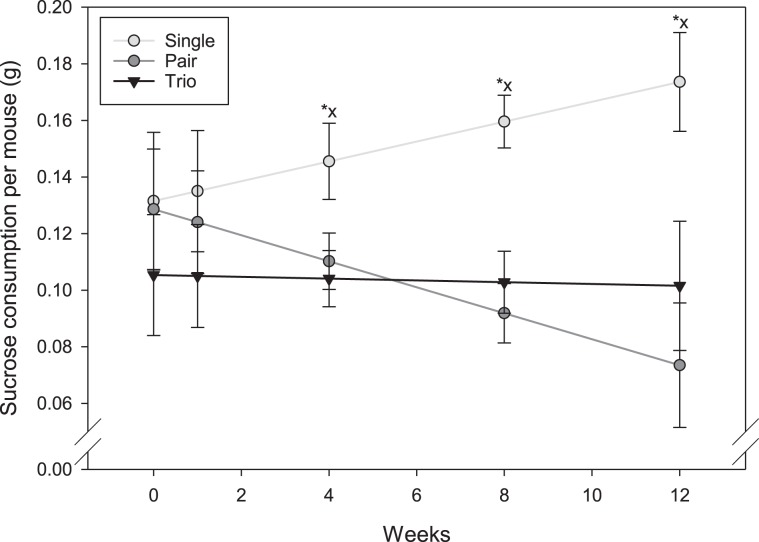
Sucrose consumption per mouse over time. Asterisks indicate significant differences pair vs single; x indicates significant differences trio vs. solitary.

Group size affected sucrose consumption per animal as the study progressed (F _1, 228_ = 5.61; P = 0.004; Fig. 2). At baseline, sucrose consumption per animal was similar between the three group sizes (P > 0.05). From week 4 to week 12 of the experiment, individually housed mice consumed more sucrose than both pair and trio housed mice (P < 0.05).

#### Nest scoring

Nest scores, in general, were high (4–5). We observed an interaction between group size and age (F _2, 581_ = 4.0; P = 0.02). Individually housed mice separated at 5 weeks built higher scoring nests than did pair or trio housed mice grouped at the same age (P < 0.05). Age of grouping and handling condition also altered nest scores (F _1, 581_ = 18.3; P < 0.001). Minimally handled mice grouped at 7 weeks built higher scoring nests than did those grouped at 5 weeks. Nest scores for scruffed mice were similar between ages (P > 0.05). Additionally, mice grouped at 7 weeks, who were minimally handled, had higher scoring nests than did those grouped at 5 weeks (P < 0.05). However, in mice grouped at 5 weeks, scruffed mice built higher scoring nests than did minimally handled mice (P < 0.05). Last, nest scores changed over the course of the study depending on handling (F _1, 581_ = 13.1; P < 0.001). Nest scores of minimally handled mice decrease during the study while nest scores of scruffed mice increased.

### Physical well-being

#### Food consumption

**Figure 3:**
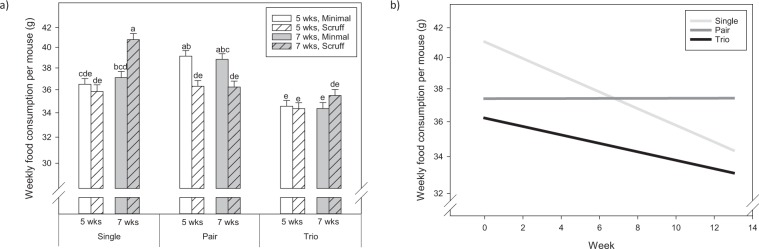
Food consumption per mouse per week. (**a**) Comparison of treatment groups. Different letters above bars indicate significant differences by post-hoc Tukey tests (P < 0.05); (**b**) Development over time. Data on the y-axis is represented as a log_10_ transformed scale but values have been backtransformed to be meaningful in the real world.

Two single housed animals showed relatively high food consumption, but no corresponding increase in body weight. No signs of food grinding or comparable behavior was observed in these animals. A significant three-way interaction between group size, age at arrival, and handling condition (F _2, 695_ = 3.6; P = 0.027; Fig. 3a) was observed. Mice, housed at 7 weeks, and minimally handled, consumed the most food per mouse (P < 0.05). Group size also affected food consumption over the course of the study (F _2, 695_ = 13.1; P < 0.001; Fig. 3b). Bonferroni corrected custom tests revealed that food consumption decreased for individually housed mice and increased for pair housed mice over the 13 weeks (F_1,695_ = 18.9; P < 0.001; F_1,695_ = 20.2; P < 0.001). However, food consumption for mice housed three per cage was consistent throughout the study, or the slope was not different from zero (F_1,695_ = 0.003; P = 0.95). The blocking factor, average body weight per cage was also significant (F_1,695_ = 5.97; P = 0.015).

#### Body weight

**Figure 4:**
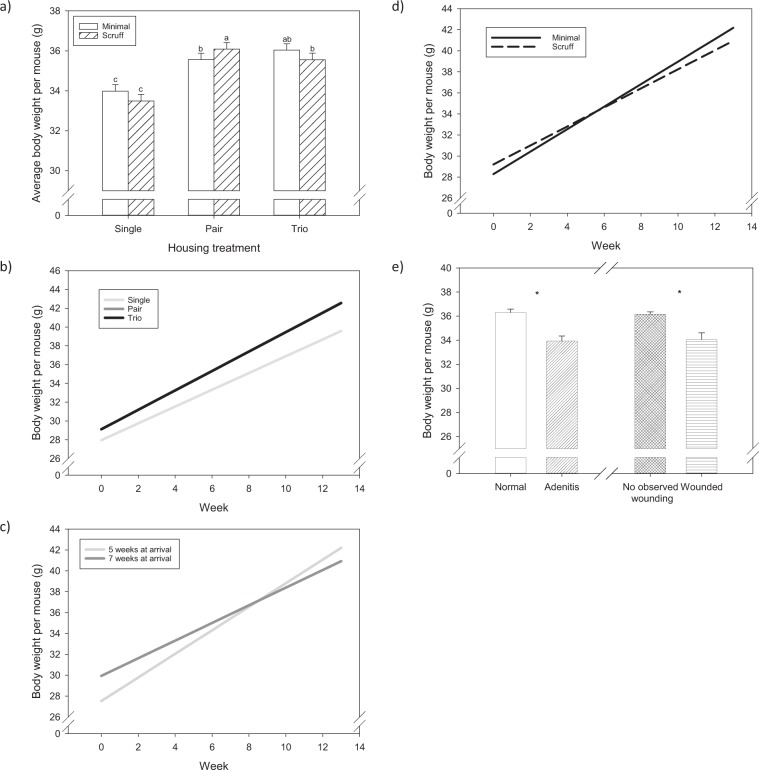
Average body weight per mouse. (**a**) Comparison of treatment groups. Different letters above bars indicate significant differences by post-hoc Tukey tests (P < 0.05); Bodyweight change per mouse over the experiment: (**b**) group size, (**c**) age at allocation and (**d**) manipulation frequency. (**e**) Body weight per mouse according to health status. Asterisks indicate significant differences.

Body weight increased continuously in all groups over the course of the study. Group size and age at grouping affected body weight (F _2,755_ = 42.2; P < 0.001). Generally, mice in larger groups weighed more (P < 0.05). However, when housed three per cage, mice grouped at 7 weeks were heavier, on average, than mice grouped at 5 weeks (P < 0.05). A significant two-way interaction between group size and handling condition was seen (F_2,755_ = 11,1; P < 0.001; Fig. 4a). Daily scruffing resulted in significantly heavier animals in pair housed mice (P < 0.05), however, no effect was seen in individually housed mice or mice housed three per cage (P > 0.05). Additionally, we observed a significant interaction between age at group allocation and handling condition (F_1,755_ = 25; P < 0.001). Scruffing had a negative effect on the body weight of mice housed together at 5 weeks (P < 0.05). There was a significant two-way interaction of week of grouping with all three main variables (group size by week: F_1,755_ = 13.7; P < 0.001; age by week: F_1,755_ = 121.8; P < 0.001; handling condition by week: F_1,755_ = 41.90; P < 0.001; Fig. 4b–d). All group size treatments increased bodyweight over the course of the study, but individual mice always weighed less than mice in other group sizes. Mice that were housed at 7 weeks were unsurprisingly heavier than those at 5 weeks. However, by about the 8^th^ week of the study, both groups of mice weighed the same. Minimally handled mice were about a gram lighter at the beginning of the study but were about a gram heavier at the end of the 13 weeks. Mice that showed signs of sickness (F_1,755_ = 39,6; P < 0.001; Fig. 4e) or had observed fight wounds (F_1,755_ = 14.1; P < 0.002; Fig. 4e) were significantly lighter than those without.

#### Fecal corticosterone metabolites

**Figure 5:**
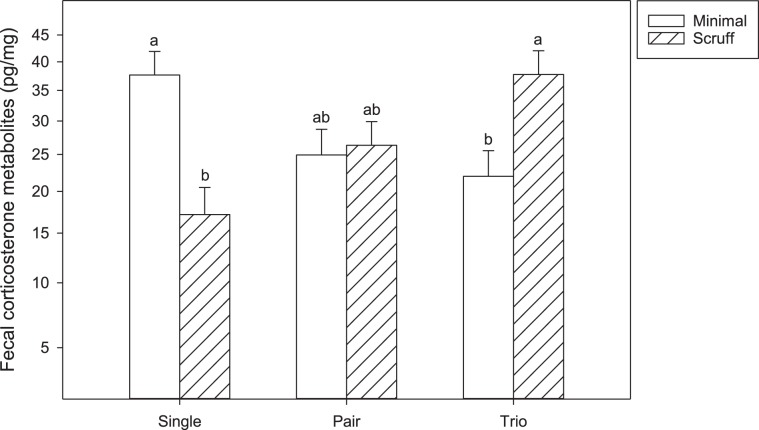
Fecal corticosterone metabolites from baseline data only. Baseline was the only time point that showed significant differences via test slices. Therefore, only baseline data has been graphed. Data are represented on the y-axis as a square root transformed scale but values have been backtransformed to be meaningful in the real world. Different letters above bars indicate significant differences by post-hoc, Bonferroni corrected, T-tests (P < 0.0055).

In general, corticosterone metabolites changed over the course of the study (F_2, 88_ = 39.1; P < 0.001). Baseline values were higher than those at week 1 and week 12 (P < 0.05). However, there was no difference between week 1 and week 12 (P > 0.05). An interaction between group size, handling, and time of grouping was found to be significant (F_4, 88_ = 3.7; P = 0.007). Using test slices in JMP, only baseline data showed differences between these values (F_5, 94_ = 4.9; P = 0.004, Fig. 5). Bonferroni corrected T-tests identified that within minimally handled cages, single housed mice had higher metabolites than trios (P < 0.0055). Single, minimally handled mice also had higher metabolite values than those scruffed (P < 0.0055). However, for trio housed mice, minimally handled mice had lower values than scruffed mice (P < 0.0055). Finally, within the scruffing condition, trio housed mice had higher metabolite values than individually housed mice (P < 0.0055).

#### Hematology

While average white blood cell count was not affected by group size, age of grouping or handling, the average red blood cell count showed a significant two-way interaction between group size and handling (F_2, 51_ = 4.4; P = 0.017). Pair housed mice that were scruffed had a higher red blood cell count than the minimally handled pair housed mice (P < 0.05). Neutrophil:Lymphocyte ratio was affected significantly by handling (F_1,53_ = 4.6; P = 0.037) with scruffed animals having lower ratios.

## Discussion

In this study, we systematically analyzed the effect of different housing conditions and husbandry procedures on the occurrence of wounding due to aggressive behavior and general mouse welfare. Male CD-1/ICR mice were housed under different conditions for 14 weeks and behavioral, clinical, endocrinological and hematological indicators were assessed during the course of the study to test our hypotheses.

The studied mice showed unexpectedly low levels of aggression, during brief clinical observations, and a low number of wounds during the 14 weeks. In fact, no animals had to be euthanized or separated due to inter-male aggression. This was confirmed by post-mortem pelt evaluation (PALS) where no severe wounding was detected. Directly after group allocation and during the first half of the observation period, fighting or fight wounds were rare and only observed after three weeks in two cages. Over time, in the second half of our observation period, when the males grew older, the observable aggression increased only slightly. Mainly pairs of mice and minimally handled animals were affected, while age at group allocation played little to no role. Analyses of the PALS support the conclusions from the visual, daily observations, with pairs and minimally handled animals showing the highest wounding scores. It is important to highlight that during short clinical observations, we only identified aggression in four cases but found 25 animals with positive PALS at post-mortem analyses. This data is important to point out since routine cage-side observations of male mice may underestimate aggression and wounding. Continuous home cage observations or observations during the dark cycle may have resulted in a more realistic picture of aggression levels.

Male CD1 mice are described as aggressive and territorial^24,40^, even when housed in sibling groups^26^. Nevertheless, we observed a relatively low level of aggression in our mice. Studies on mouse aggression often use the resident intruder test^7,32^ which measures extra-group aggression (i.e. mediated aggression). In our study, we evaluated the results (wounding) assumed to result from excessive escalated aggression in the home cage. Intra-group and extra-group aggression are not necessarily related and can have different underlying motivational states and consequences in mice^26,33^. Additionally, it is known that nest building material can decrease inter-male aggression^29^, specifically when transferred during cage change^6,28^. Our standard housing conditions might therefore have already reduced aggressive behaviors in our study mice. This effect might have been amplified by testing relatively small group sizes. Several studies indicate that a group size of three correlates with more social stability and less aggression compared to larger group sizes^26–28,30^. Surprisingly, even smaller group sizes (i.e. pairs) did not further reduce aggression, but increased the incident of bite wounds in our study. Van Loo and colleagues postulated that groups of three are ideal because one male is the obvious “dominant”, another serves as the middle “peace keeper” and the third as the submissive animal^30^. Our results might provide some support but a detailed behavioral analysis would be required to truly substantiate these claims.

In addition, handling had a distinct effect on wounding. Aggression was assumed to increase with disturbances^9,24,36^. Surprisingly, in our study, mice that were handled more frequently showed less wounding and were, at least when pair housed, heavier than mice handled less frequently. The negative physiologic effects of stress are known to be mitigated when the stressor can be predicted or controlled^41^. Daily scruffing, due to its frequency, probably led to more predictability than weekly handling.

On the other hand, the effect of age on aggressive behavior is unclear. While Bartolomucci found increased aggression^25^, several studies state that group allocation before sexual maturation results in low levels of aggression^9,42,43^. Mice reach sexual maturity anywhere between 5 to 7 weeks of age^44^. Perhaps if we had chosen to test a larger age difference at the time of allocation, we might have found more pronounced differences. However, our aim was to test relevant ages of group allocation that could be practically used by researchers.

Despite the effects of housing conditions on male aggression, we also found differences in several parameters that have effects on animal welfare. In general, mice that were wounded or sick (primarily preputial adenitis) had reduced body weights, which is an expected sign of physical impairment. Mice in our study housed three per cage ate the least, but also had the highest body weights. This feed:gain ratio could suggest potentially less social stress in trio housed mice compared to pair housed mice, which is also supported by fewer incidents of fighting and lower PALS. Body weight increased in all mice over the course of the study, which closely aligned with a typical growth curve of CD1 mice^45^. However, single housed mice were fell toward the lower end of the weight range.

The feed:gain ratio of trio housed mice may also suggest less thermal stress in group housed animals compared to individually housed animals. Solitary mice consumed more food, as well as more sucrose and built more complex nests, but gained less weight than group housed mice. Therefore, there are clear indicators of thermal stress in individually housed mice even though they were provided with the recommended amount of nesting material (8–10 g)^46,47^. It is not completely surprising that we saw evidence of cold stress in individually housed mice since the recommended amount of material is based on data from trio housed mice. Further, 10 g may not be sufficient to completely eliminate cold stress in some mice under typical laboratory temperatures^47^. Huddling with conspecifics is a very basic behavioral mode of thermoregulation. In hamsters, huddling conserves energy and reduces food consumption when thermally challenged^48^. Cold stress has also been previously documented in other studies with mice. For instance, Nagy *et al*. reported that individually housed B6 males were smaller than group housed males^49^.

Behavioral indicators, such as nest complexity and sucrose consumption, have been proposed as general measures of mouse well-being. For instance, decreased nest complexity or nests that generally appear more open and flat can be an indicator of various welfare issues, such as pain^50,51^, infection^52,53^, handling stress^52,54^ or male aggression^9,55^. In a mild social defeat model using C57BL/6JJmsSlc mice, males successfully built nests, but nest building was severely delayed compared to control mice^56^. Also, lower levels of sucrose consumption may indicate anhedonia and therefore reduced well-being. Sucrose consumption is usually assessed via ingestion of a sucrose solution, but the use of solid sucrose, as used in our study, is a validated alternative^57^. We observed no difference in sucrose consumption in group housed animals, but consumption was increased in individually housed mice, which seems to correlate with a parallel decrease in food consumption. Individually housed mice may have preferred the source of high caloric content to fulfill energy demands due to thermal stress. Nest complexity and sucrose consumption may be valid measures of well-being, however in our study, these measures might have been affected by cold stress. It is likely that individually housed mice modulated their need for warmth via more complex, better insulated nests and compensated their higher energy needs with calorie rich food items. Therefore, when using these tests to assess animal welfare, thermoregulatory needs must be carefully considered^54^.

As a measure of acute and chronic stress we also assessed fecal corticosteroid metabolites and neutrophil-lymphocyte ratio. Corticosterone or corticosteroid metabolite concentrations are commonly used to measure stress in laboratory animals^58^ and the neutrophil-lymphocyte ratio has been proposed as a potentially better indicator of chronic stress. Elevated serum corticosterone concentrations, but not neutrophil-lymphocyte ratios, have been associated with acute stress exposure, whereas elevated neutrophil-lymphocyte ratios have been associated with chronic stress exposure in male Sprague Dawley rats^59^ and in male and female C57BL/6 N mice^60^. No measurable effect of housing condition on stress levels of mice were observed in our study. Corticosterone metabolite levels were significantly higher during baseline measurements compared to week 1 and week 12 in all groups. Thus, we likely measured the stress effect of transport from the animal breeder to our facility as well as stress related to the animal identification procedure shortly after arrival. The low corticosterone metabolite levels at week 12 suggest relatively low stress in our animals independent of treatments. While corticosterone levels have been successfully used to assess stress in mice, it is not clear if elevations in levels also correlate with increased aggression in male mice. Several studies have shown no difference in corticosterone levels between male and female mice housed in smaller or larger groups^12,30,61^ while other studies have reported lower levels in individually housed males^13^. In our study, the neutrophil-lymphocyte ratio was lower in scruffed animals, again suggesting a positive effect of more predictable handling procedures but did not differ for group size or age at allocation. Likewise, while white blood cell count was not affected, red blood cell count was slightly higher in scruffed pairs. Further, no differences were seen between individual and group housed mice. These standard toxicology read-outs were therefore not affected by housing conditions in our study.

In summary, laboratory mice are social animals and are highly motivated to interact with one another^1^ and several studies show distinct effects of individual housing^15,16,62,63^. Nevertheless, aggression has been described as the second most common reason for premature death or euthanasia in male laboratory mice^64^. Individual housing due to male aggression is a welfare problem which may influence data variability^7^, increase animal housing costs, space allocation, and other resource requirements. Therefore, identifying housing and husbandry conditions that reduce inter-male aggression is necessary in order to move to successful group housing as a standard. Our data suggest that housing male mice in groups of three per cage is a practical and economical solution to improve animal welfare by reducing the likelihood of wounding due to inter-male aggression and eliminating the effects of cold stress experienced by individually housed mice.

## Methods

### Animals and husbandry

All animal studies were reviewed and approved by AbbVie’s Institutional Animal Care and Use Committee. All procedures adhere to the Guide for the Care and Use of Laboratory Animals. Animal studies were conducted in an AAALAC accredited program and veterinary care was available to ensure appropriate animal care. Male CD-1/ICR (120) mice were obtained from a commercial breeder (Charles River Laboratories, Raleigh, USA, Rooms 1 and 9) and were 5 or 7 weeks of age upon arrival. Animals were free of common mouse pathogens and hygiene level was monitored with a sentinel program and additional environmental swabs.

The animals were kept in Allentown IVC mouse drawer style cages (7115 (7.5″ × 11.75″ × 5” cage (67in²)), in Ecoflo racks (supply and exhaust set to 60 ACH) with Edstrom stainless steel automated watering. Cages contained aspen bedding (Coarse grade Sanichip, P.J. Murphy Forest Products) and two 4-gram pucks of nesting material (Bed R’ Nest, The Anderson’s, Brown/Kraft). Mice were fed ad libitum (2014 Global Rodent Diet, Envigo) and had unrestricted access to drinking water (reverse osmosis, hyper-chlorinated, ~1ppm). The light/dark cycle in the room consisted of 12/12 h with artificial light (On: 6AM, Off: 6PM). The room temperature was set to 72 °F (+/− 2 degrees, avg. ~72.8 °F) with a relative humidity of 30–70% humidity (avg. ~55%). The room was monitored with Edstrom Watchdog System. A complete cage change was conducted at the beginning of every week. During the cage change, used, but unsoiled, nesting material was transferred from the dirty cage to the new, clean cage. Animals were handled by the tail for all procedures.

### Group allocation

**Figure 6:**
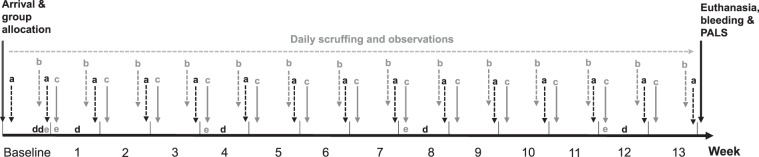
Experimental timetable. a = body weight measurements, b = cage change, food intake, c = nest score, d = sucrose consumption, e = fecal samples.

Upon arrival, animals were tail tattooed for identification and randomly allocated to one of 12 groups (see Table 1), balanced across age at arrival (5 or 7 weeks of age), group size (single, pair or trio), and handling condition (daily scruffing or minimal handling, only at cage change). This resulted in five cages per group. Randomization was performed using a spreadsheet designed by AbbVie for randomization purposes (total randomization, no group concealment at randomization). Mice were left undisturbed to acclimate to the new environment after shipment for one week before baseline measurements were collected (see Fig. 6). Half of the animals arrived two weeks before the others (1. cages 1–36, 2. cages 37–60; balanced across all treatment groups) so that data could be collected in a timely manner at the end of the 13 weeks.

**Table 1:** Treatment groups (5 cages per group).

Group	Group size	Age at arrival	Handling treatment	Total number animals
1	1	5	No scruff	5
2	1	5	Scruff	5
3	1	7	No scruff	5
4	1	7	Scruff	5
5	2	5	No scruff	10
6	2	5	Scruff	10
7	2	7	No scruff	10
8	2	7	Scruff	10
9	3	5	No scruff	15
10	3	5	Scruff	15
11	3	7	No scruff	15
12	3	7	Scruff	15

Mice assigned to the scruffing condition received the following handling procedure: the tail was pressed with the 4th and 5th finger to the palm of the experimenter’s hand, while thumb and forefinger grasped the scruff of skin at the back of the mouse’s skull. The mouse was held like this for several seconds before it was released back into its home cage.

### Measurements

Measurements were performed at the cage level, except for body weight measurements, daily observations, hematology and necropsy which were conducted for every animal but were averaged per cage for analysis. Several non-blinded, male and female animal care technicians performed measurements.

### In life measurements

Daily observations: Animals were observed daily at 12 pm, before the scruffing procedure, to assess fighting (e.g. biting or rough and tumble fighting) and the number of animals out of the nest. Since our goal was to determine whether aggression was generally observed by husbandry staff, reliability was not assessed for this observation. This time was chosen to coincide with when nests should be scored (nest shape peaks 7–12hrs after the lights come on^50^) to reduce disruptions and improve data collection efficiency. Additionally, mice are generally resting in the nest at this time. Thus, social isolation outside of the nest may have been indicative of poor welfare (e.g. illness^65^). A visual health check of animals was performed, including recording changes in overall body condition (hair coat, urine staining, mouth/malocclusion, extremity issues) as well as the occurrence of bite/fight wounds. Any open wounds from fighting were determined as humane endpoint criteria. Cages were opened and animals were only picked up if further clinical assessment was needed.

Food consumption: During weekly cage change, food remaining in the food hopper was weighed and when needed, the hoppers were refilled and re-weighed.

Body weight: Body weights of every mouse were measured at the end of every week.

Nest scoring: Nests were scored every week, three days after cage change and the day before body weights were measured. This day was chosen to avoid the destruction of nests due to husbandry or experimental procedures. Scores were recorded between 12:00 and 13:00 using a scale from a previously published protocol^55,66^. In brief, four walls of the nest were given a 0–5 score which was averaged together to provide the cage a nest score. A score of 0 was assigned to cages where nesting material was present but left undisturbed, a score of 1 was assigned when the nesting material was manipulated but no nest site could be identified, a score of 2 was assigned when the nest was flat, a score of 3 was assigned when the nest was cup-like, a score of 4 was assigned when the nest was an incomplete dome, and a score of 5 was assigned when the nest was a complete and enclosed dome. Nest scores from week two and eight from animals in the first replicate and week four and ten of animals in the second experimental replicate were discarded due to low data reliability from a single technician.

Sucrose consumption: Sucrose consumption was measured two times during baseline week and once in week one, four, eight and twelve. Cages were provided with one sugar cube per animal at 06:00 am in their home cages. The cubes were weighed before and after 30 minutes^67^.

Fecal corticosterone: Feces were collected with forceps from the clean cage between 08:00 and 09:00, 2 to 3 hours after cage change, the day before the start of the study (baseline) as well as at weeks one, four, eight and twelve. Samples were stored in a 15 ml conical tube in a −80 freezer. Fecal corticosterone metabolites were analyzed by Arbor Assays (Ann Arbor, MI USA). The methods of this analysis were the same as those used by LaFollette *et al*.^68^.

### Post-mortem measurements

Blood collection and hematology: Blood was collected via cardiac puncture after confirming plane of anesthesia had been reached by checking for loss of reflex following exposure to CO_2_ (30% CRR). Blood (250–500ul) was collected using a 1 ml syringe with 25 gauge needle; the blood was placed into EDTA tubes and gently mixed. The blood was maintained at room temperature until further processing, shortly after collection. Hematology samples were evaluated on the Siemens ADVIA 2120 automated hematology analyzer (red blood cell count, neutrophil count, and lymphocyte count). The neutrophil-lymphocyte ratio was hand calculated.

Necropsy and Pelt Aggression Lesion Score (PALS): Animals were inspected after death to record any signs of injury or disease. For PALS assessment, animals were placed in dorsal recumbency and a ventral midline incision was made from pubis to mandible and the skin was freed from the carcass. The skin was then placed on a dissection board, subcutis facing upward and then photographed. Photos were assessed by one author (PJ) who was trained by an experienced colleague and fully blinded to experimental condition. Before scoring, a set of pictures (25%) were scored by both the experimenter and the trainer; discrepancies between observer scores were rare, but were discussed and resolved before the actual scoring was performed. To assess wounding, a 3 × 3 grid was sized and overlaid on top of each pelt photo. The grid was aligned from the base of the neck to the base of the tail, and limits of the pelt midsection to each side. For each of the 9 areas of the grid, the amount of the square that could actually be scored was estimated as 0–25%, 26–50%, 51–75%, or 76–100%. Portions of the section were considered not scoreable if the pelt did not completely fill the square. This designation was intended to give more weight to sections that were fully observable. Each of the nine sections were then scored for injury based on the severity scale published^69^. Finally, the PALS score for each region was calculated as: PALS region score = Severity Score x Area Score × 0.25. Hemorrhaging was observed in all mice from the first block of animals around the neck and shoulders when the mice were pelted (potentially from cervical dislocation being used as a secondary method of euthanasia) which made it difficult to distinguish from aggression related hemorrhaging. This was only done with the first block of animals, which included mice from all treatment groups. Additionally, several single housed mice (n = 3) showed small wounds that looked like bite wounds on the lower back area, leading to several false positives in individually housed mice. Therefore, the three anterior sections, shoulder region on the pelt that contained the hemorrhaging were excluded and only the six lower regions from the mid-back and posterior sections were used in our PALS analyses. These areas have been shown to be the most common areas for the majority of aggression related wounding using the PALS scoring scheme^69^.

### Statistical analyses

All data collected were statistically analyzed as a mixed model GLM in JMP 13 (SAS, Cary, NC). The assumptions of a GLM (normality of error, homogeneity of variance, and linearity) were confirmed graphically post hoc and appropriate transformations made, if necessary^70^. Factorials are specifically designed to maximize power and reduce sample size because they incorporate, account for, and eliminate unwanted variance. The use of factorials and repeated measures designs are commonly used as a means of reduction and refinement. This approach allows us to test for general effects of each variable, and also look for interactive effects with greater power than non-factorial analyses^70^. In addition, this design allowed us to test our experimental conditions while controlling for body weights and repeated measures per cage for some measures. The experimental condition was applied to the cage of mice; therefore, our experimental unit was the cage. When multiple measures were derived from mice in the same cage (e.g. body weight) they were processed to a mean to assess the overall effect of condition. Cage was treated as a fixed variable. P was considered significant if <0.05 and all data is presented as least square means ± standard error (LSM ± SE). PALS was considered our main outcome variable. Since some data was lost due to various reasons, please refer to Table S1 to see which combination of treatments were not completely balanced.

The final model is presented for each outcome variable.

#### PALS

A full factorial was originally tested but due to an unbalanced dataset, non-significant interactions were removed from the final model. Data was log_10_ transformed for normality. Significant effects were tested using post-hoc Tukey tests.

log_10_ (Avg PALS per cage + 1) = AgeTrt + GrpSize + HandlingTrt

#### Food consumption

In addition to the basic factorial model, cage average body weight was included as a covariate. Blocking factors of illness and signs of fighting were also tested but were not significant and were therefore dropped from the final model. Significant effects were tested using post-hoc Tukey tests

Avg Food consumption per cage = Cage[Group size,Age,Treatment] + Group size + Age + Treatment + week + Group size*Age +  + Group size*Treatment + Group size*week + Age*Treatment + Age*week + Treatment*week + Group size*Age*Treatment + body weight cage average

#### Body weight

The body weight analysis also included blocking factors (Y/N) of whether a preputial gland abscess or other illness was observed and whether external fight wounds were observed (Y/N) each week. Body weight data was also log_10_ transformed to meet the assumptions of normality. Significant effects were tested using post-hoc Tukey tests.

log_10_(Average body weight) = Cage[Group size,Age,Treatment] + Group size + Age + Treatment + week + Group size*Age + Group size*Treatment + Group size*week + Age*Treatment + Age*week + Treatment*week + Group size*Age*Treatment + illness + fight wounds observed

#### Nest scoring

Significant effects were tested using post-hoc Tukey tests.

Nest score = Cage[Group size,Age,Treatment] + Group size + Age + Treatment + week + Group size*Age + Group size*Treatment + Group size*week + Age*Treatment + Age*week + Treatment*week + Group size*Age

#### Sucrose consumption

In addition to the basic model, cage average body weight was included as a covariate. Significant effects were tested using post-hoc Tukey tests.

Average sucrose consumption = Cage[Group size,Age,Treatment] + Group size + Age + Treatment + week + Group size*Age + Group size*Treatment + Group size*week + Age*Treatment + Age*week + Treatment*week + Group size*Age*Treatment + body weight cage average

#### Fecal corticosterone metabolites

Fecal corticosterone metabolite data were tested similar to the factorial stated above but only baseline, week 1, and week 12 were analyzed. Due to collection and ELISA evaluation issues some data was lost (see Supplementary Table 1 for the number of data points per treatment combination). Repeated measures data (pg/mg feces) was square root transformed for normality. Significant effects were tested using post-hoc Tukey tests and Bonferroni corrected test slices in JMP.

Sqrt(corticosteronemetabolites) = Cage[AgeTrt,GrpSize,HandlingTrt] + AgeTrt + GrpSize + HandlingTrt + Week + AgeTrt*GrpSize + AgeTrt*HandlingTrt + AgeTrt*Week + GrpSize*HandlingTrt + GrpSize*Week + HandlingTrt*Week + AgeTrt*GrpSize*HandlingTrt + GrpSize*HandlingTrt*Week

In a separate analysis on week 12 data only, we tested whether PALS and illness at necropsy altered metabolite levels.

Week12Corticosteronemetabolites = AgeTrt + GrpSize + AgeTrt*GrpSize + HandlingTrt + AgeTrt*HandlingTrt + GrpSize*HandlingTrt + AgeTrt*GrpSize*HandlingTrt + ObsSickatEnd + AvgOfPAL Score

#### Hematology

Due to a few lost data points with the hematology samples and samples being pooled per cage, interactions were dropped if not significant and the analyses did not include the blocking factor of cage. The blocking factor of illness was tested for all hematology measures but was not significant and therefore not included in the analysis. Significant effects were tested using post-hoc Tukey tests.

Neutrophil: Lymphocyte data was Box-Cox transformed to meet the assumptions of normality.

N:L = AgeTrt + GrpSize + HandlingTrt

White Blood Cell count

WBC = AgeTrt + GrpSize + HandlingTrt

Red Blood Cell count

RBC = AgeTrt + GrpSize + HandlingTrt + GrpSize*HandlingTrt

## Supplementary information

Supplementary table 1.

Supplementary Dataset

## Data Availability

The data used in this study is available as supplementary information.
