# Supplementary material for: The effect of group size, age and handling frequency on inter-male aggression in CD 1 mice
Source: Sci Rep. 2020 Feb 10;10:2253. doi: 10.1038/s41598-020-59012-4 (PMC7010790; doi:10.1038/s41598-020-59012-4)
Supplement: Supplementary file 1 — Supplementary table 1. [file 41598_2020_59012_MOESM1_ESM.docx]

# The effect of group size, age and handling frequency on inter-male aggression in CD 1 mice

Authors: Paulin Jirkof^1*^, Natalie Bratcher^2^, Letty Medina^2^, Donna Strasburg^3^, Paige Ebert^3^, Brianna N. Gaskill^4^

^1^ Division of Surgical Research, University Hospital Zurich, Switzerland

^2^ Office of Animal Welfare, AbbVie, USA

^3^ Comparative Medicine, AbbVie, USA

^4^ Department of Animal Science, Purdue University, USA

* Corresponding author: [paulin.jirkof@usz.ch](mailto:paulin.jirkof@usz.ch)

**Supplementary table 1**: Compilation of the number of data points included in our statistical analyses. Combinations which are missing data were omitted from the analysis for reasons included in the manuscript. End measures include a single data point per cage: PALS, N:L, WBC, and RBC. Repeated measures, where data was collected multiple times over the experiment include: Food consumption, body weight, nest score, sucrose, and fecal corticosterone metabolites.

| Group Size | Handling | Age | PALS | Food consumption | Body Weight | Nest Score | Sucrose | Fecal Cort. Metab | N:L | WBC | RBC |
| --- | --- | --- | --- | --- | --- | --- | --- | --- | --- | --- | --- |
| 1 | Minimal | 5 | 5 | 65 | 70 | 55 | 25 | 13 | 5 | 5 | 5 |
|  |  | 7 | 5 | 64 | 70 | 55 | 25 | 13 | 5 | 5 | 5 |
|  | Scruff | 5 | 4 | 51 | 56 | 44 | 20 | 12 | 4 | 4 | 4 |
|  |  | 7 | 5 | 65 | 70 | 55 | 25 | 14 | 5 | 5 | 5 |
| 2 | Minimal | 5 | 5 | 65 | 70 | 55 | 25 | 14 | 5 | 5 | 5 |
|  |  | 7 | 5 | 65 | 70 | 55 | 25 | 14 | 5 | 5 | 5 |
|  | Scruff | 5 | 5 | 65 | 70 | 55 | 25 | 15 | 5 | 5 | 5 |
|  |  | 7 | 5 | 65 | 70 | 55 | 25 | 15 | 5 | 5 | 5 |
| 3 | Minimal | 5 | 5 | 64 | 68 | 54 | 24 | 14 | 5 | 5 | 5 |
|  |  | 7 | 5 | 65 | 70 | 55 | 25 | 14 | 5 | 5 | 5 |
|  | Scruff | 5 | 5 | 63 | 67 | 52 | 24 | 14 | 4 | 4 | 4 |
|  |  | 7 | 5 | 65 | 70 | 55 | 25 | 15 | 5 | 5 | 5 |
